# Cardiometabolic Clinics: Is There a Need for a Multidisciplinary Clinic?

**DOI:** 10.3389/fcdhc.2022.880468

**Published:** 2022-06-06

**Authors:** Yosef Manla, Wael Almahmeed

**Affiliations:** Heart and Vascular Institute, Cleveland Clinic Abu Dhabi, Abu Dhabi, United Arab Emirates

**Keywords:** cardiometabolic, clinics, multidisciplinary, diabetes, obesity

## Introduction

Approximately half a billion people live with diabetes mellitus (DM) worldwide. (1) Additionally, the prevalence of both DM and obesity, a major risk factor for type 2 DM, has doubled by 2015 compared to 1980 (2, 3). Given its high burden of morbidity and complications, DM has been ranked the ninth leading cause of death globally in 2019 (4). Furthermore, diabetes mellitus and obesity co-exist with a nexus of conditions, among others, hypertension, dyslipidemia, coronary artery disease (CAD), heart failure (HF), resulting in what is referred to as cardiometabolic syndrome (CMS) with a recent analysis revealing that half of type 2 DM patients suffers from at least three cardio-renal-metabolic conditions (5). On the other hand, patients with DM are over-represented in cardiology outpatient clinics, coronary care units, and cardiovascular disease (CVD) remains the leading cause of death within this population. (6) Besides, abnormal glucose regulation is underdiagnosed in the CVD population; a large observational study in Europe has demonstrated that patients with CVD had abnormal glucose regulation in about two-thirds of the cases (7). This situation with multiple systemic conditions impacts the clinical course, quality of life, and long-term survival of patients with DM. Therefore, there is a substantial necessity for cardiometabolic multidisciplinary clinics (CMC) as a forum of collaboration for multiple specialities to achieve optimal CMS management.

## Cardiometabolic Health Care Economics

DM exhausted health care resources in 2019 with an expenditure of USD 760 billion (1). In addition, the effect of suffering from multiple simultaneous cardiometabolic morbidities on health expenditure has been widely researched with estimated projections that obese patients with three cardiovascular risk factors (CRF) have 14-fold annual health costs compared to obese patients with no CRF (USD 12,190 vs. 838) (8). Another meta-analysis of 383,420 individuals with one or more components of CMS (hypertension, diabetes, dyslipidaemia, adiposity) revealed an adjusted total annual health care cost of USD 5,564 per individual in patients with one component of CMS, while the cost of care for those with four components came to USD 12,287 (8). Nevertheless, early detection is essential in improving short- and long-term outcomes; it worth to be mentioned that in a recent analysis of the INTEGRATE randomised controlled trial, the implementation of primary care-based cardiometabolic risk prevention programs failed to show cost-efficacy in the long-term (9). In the light of the above-mentioned economic burden of CMS, there remains a need for a new comprehensive model that will foster more effective interventions with sustained health effects at reasonable costs.

## Challenging CMS Patient Management in a Fragmented Consultative Model

In the current dominating consultative model of care, ease of access to health services in networked systems has facilitated patient referrals. Subsequently, patients see more than one healthcare professional, resulting in a significant economic burden reflected by high reimbursement rates (10). Such impact is more pronounced in CMS, in which several obstacles can be identified. First, there is no centralisation of care associated with one physician or clinic, as patients may rotate between multiple providers (primary care practitioner, endocrinologist, cardiologist), with a considerable overlapping in CRF management (11). This leads to interference in deciding the optimal drug class, dosing, drug interactions and may confuse patients receiving misaligned recommendations from multiple providers, resulting in ineffective follow-ups, and even eroding trust, especially when inter-provider communication is lacking (11).

Furthermore, approaching such patients from a single speciality perspective and not overviewing the complex picture may result in underestimating adverse outcomes and long-term complications of DM, as the risk of CVD is increased by two to four folds (12). When it comes to acute coronary syndrome and HF patients, DM and co-existing comorbidity management in a patient-centred fashion is vital. Other essential aspects of cardiometabolic therapy, such as lifestyle modification and psychological counselling, are inadequately addressed in the conventional model of care (11, 13). As a result, CMS patients receive fragmented care featured by redundant diagnostics at higher costs and are at risk of drug-drug interactions and, most importantly, adverse CVD events (11).

## The Role of Cardiometabolic Clinics in Eliminating Current Practice Gaps

Despite the strong evidence supporting the efficacy of current CV preventive therapies in avoiding adverse events and improving survival, there is still a lack of adherence to these agents, causing inadequate control of major CRF (14, 15). Evidence has revealed that therapeutic targets of the major three components of CMS (hypertension, hyperlipidemia, diabetes mellitus) were simultaneously achieved only in 7-19% of the patients (16, 17).

On health care professional level, a recent USA based data describing how frequent type 2 DM patients visit their health providers showed a two-fold higher rate of cardiology follow-ups vs. endocrinology reviews among type 2 DM patients and that increased even up to 4 folds in CMS patients, highlighting the possible contribution that cardiology specialists can make in prescribing guideline-directed therapies, especially, those with cardio-metabolic-renal merits (18). However, this contribution is still limited in prescribing FDA-approved sodium-glucose co-transporter (SGLT-2i) inhibitors since cardiologists are responsible for only 5% of prescriptions, according to a recent retrospective study in Massachusetts, USA (19). A recent study featured a 3-fold increase in the prescription of glucagon-like peptide-1 receptor agonists (GLP-1RA) in the USA between 2014 and 2019; however, cardiologists contributed the least to this increase (<1% each year) (20). Another USA insurance database analysis highlighted that administration of antihyperglycemic drugs with CV benefits (SGLT-2i, GLP-1RA) has increased between 2014 and 2019 for CVD patients, yet underutilised, and these drug applications were more likely in younger with higher socioeconomic status compared to administration of metformin (21). Overall, the current adoption of cardio-beneficial glucose-lowering agents is limited, and <20% of patients with DM and atherosclerotic cardiovascular burdens were prescribed such agents, according to a large cohort study (22). On top of that, many studies have demonstrated the cost-effectiveness of these new agents; according to a recent systematic review, these new antihyperglycemic agents were reported to be cost-effective in 26 of 30 studies compared with insulin, and 13 of 15 studies compared with sulfonylureas (23). In patients with HF, a recent study reported that dapagliflozin provided an intermediate value (mainly driven by a reduction in cardiovascular mortality) compared to the standard of care which includes appropriate treatment with angiotensin-converting enzyme inhibitors, angiotensin receptor blockers, or sacubitril-valsartan plus a β-blocker (24). These underutilised and cost-effective agents emphasise the importance of reconsidering the current siloed care model and a shift to a multidisciplinary clinic.

Preliminary data regarding the initiation of such multidisciplinary clinic have revealed that SGLT-2 inhibitors or GLP-1 agonists were initiated/up-titrated in half of the patients, resulting in an effective reduction in HbA1c levels in one-third at the follow-up visit (25).

Another experience of a developing cardiometabolic centre of excellence in the USA has demonstrated high efficacy within a short timeframe in comparison to the conventional model of care, reflected by a higher rate of guideline-directed medical therapy reception, defined as a high-intensity statin, antiplatelet or anticoagulant, ACE inhibitor/ARB, and either SGLT-2i or GLP-1RA (41.1% vs. 2.3%). A higher rate of administration of the following was experienced, including ACE inhibitor (30.2% vs. 9.1%), high-intensity statins (86% vs. 77.7%), and SGLT-2i or GLP-1RA (96.1% vs. 25.7%) in patients managed at the CMC as compared to propensity-matched group managed in the conventional care settings (15). In addition, patients enrolled in the CMC had a greater reduction of weight (−10.9 vs. −1.5 lbs, p<0.001), HbA1c (−0.5% vs. −0.2%, p=0.02), systolic blood pressure (−3.6 vs. +1.4 mmHg, p<0.01), LDL level (12.1 vs. −2.8 mg/dL, p<0.01), and total daily insulin dose (−31.6 vs.+1.1 units, p<0.001) as compared with the conventional care group (15).

The complexity of managing obesity, a chronic and relapsing disease that impairs metabolism and CV health, and challenges traditional primary care practices that have been established to treat simple or less complicated conditions, necessitates the need for more efficient multidisciplinary interventions (26, 27). In the view of lifestyle interventions efficacy in obesity and associated CRF management, guidelines strongly recommend a combination of lifestyle interventions and medical treatment for patients who are overweight (26, 28–30). A recent cluster-randomized trial by Höchsmann et al. compared obesity intensive lifestyle interventions vs. usual care. Patients who received intensive lifestyle interventions lost more weight at 24 months (mean difference, −4.51% [95% CI, −5.93 to −3.10]; p<0.01), achieved better fasting glucose control at 12 months (mean change, −7.1 mg/dL [95% CI, −12.0 to −2.1]; p<0.01), and had higher levels of high-density lipoprotein cholesterol at 24 months (mean difference at 24 months: 4.6 mg/dL [95% CI, 2.9–6.3]; p<0.01) in comparison to those receiving standard care (30). However, the rate of long-term adherence to such lifestyle modifications is still low (26). Other lifestyle modifications, such as smoking cessation programs, have proven short-term efficacy in improving glycemic control and CRF in patients with type 2 DM (31).

Dietary patterns such as Mediterranean or DASH (dietary approach to stop hypertension) diet have been widely advocated and are associated with superior control of CRF (32). Additionally, recently, medical nutrition therapy (MNT) interventions have been proven effective in patients with type 2 DM (33). Given the limited access to effective lifestyle interventions in the current model, offering lifestyle counselling in a multi-speciality clinic will facilitate the implementation of such strategies and surely improve patient outcomes (13).

Finally, an important implication of the multidisciplinary model in the CMC is to enhance screening and treatment of obstructive sleep apnea (OSA), given its high prevalence among cardiometabolic patients (∼60%), the evidence of improved patient-centred outcomes and quality of life with OSA treatment in patients with CVD, and last but not least, OSA independent association with high levels of glucose and triglyceride levels in addition to markers of inflammation, arterial stiffness, and atherosclerosis (34, 35).

## The Multidisciplinary Cardiometabolic Clinic Model

The desired CMC consists of multidisciplinary staff members, including cardiologists, endocrinologists, clinical pharmacists, specialised nursing staff, nutritionists, exercise physiologists, behavioural specialists, and genetic counsellors. Nurse practitioners with dedicated training in the field play a crucial role in the CMC hence being responsible for surveillance for CRF, electronic medical record follow-up, regular vital parameter assessment, discussing laboratory and imaging outcomes with the patients, and supporting physicians coordinating inter-disciplinary therapeutic plans and effective appointment scheduling (32). Evidence revealed that diabetes and CVD care quality provided by specialised nurse practitioners was comparable to physician-based care, and it is established that a nurse navigator role is essential for the success of CMC (15, 36).

**Figure 1:**
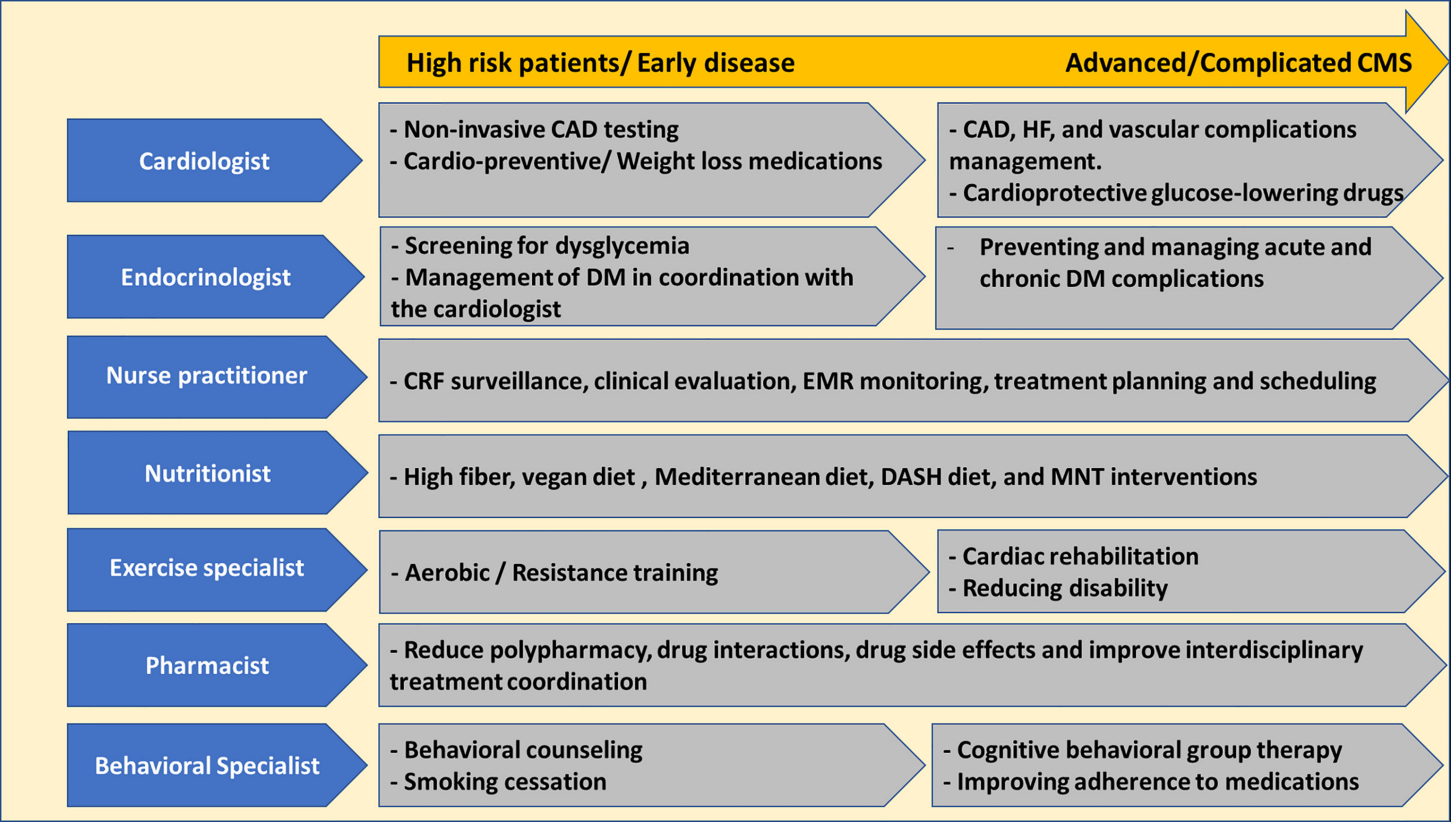
Multimodal services offered at the Cardiometabolic Clinic. CAD, coronary artery disease; CMS, cardiometabolic syndrome; CRF, cardiovascular risk factors; DASH, dietary approach to prevent hypertension; DM, diabetes mellitus; EMR, electronic medical records; MNT, medical nutrition therapy.

A multitude of services can be provided in the CMC to address risk factors, diagnose, perform multiple types of prevention and manage these patients depending on disease stage and therapeutic goals (**Figure 1**) (32, 37). In case the patient is at high risk or in the early disease stage, the care would include non-invasive CAD testing (echocardiography, stress testing, and coronary computed tomography), cardio-preventive and weight loss medications administration, behavioural counselling in addition to diet and lifestyle interventions (high fibre/vegan diet, aerobic training and smoking cessation) (32, 37, 38). The care for advanced CMS patients would extend to managing CAD and HF, improving quality of life, reducing symptoms, and disability burdens. In addition, careful clinical assessment, avoiding over-medicalization, and unnecessary harmful interventions are paramount.

## Challenges and Future Aims

Currently, the successful implementation of the CMC model is still limited. Financial costs, an excessive number of patients, and a shortage of specialised health care professionals are among the main obstacles towards the enforcement of the CMC model (11). Therefore; several strategies are suggested to enhance CMC adoption, starting by promoting for the success of such programs around the world to replicate further and implement such strategies, featuring cardiometabolic medicine on all levels of medical training (medical school, residency, fellowships). An additional layer of postgraduate development courses in cardiometabolic medicine or even board certification in cardiometabolic and lifestyle medicine is warranted by professional societies such as the American Diabetic Association and the American College of Lifestyle Medicine for health care providers to update the knowledge on this rapidly evolving medical field (39).

## Author Contributions

YM and WM joint authors. All authors contributed to the article and approved the submitted version.

## Conflict of Interest

The authors declare that the research was conducted in the absence of any commercial or financial relationships that could be construed as a potential conflict of interest.

## Publisher’s Note

All claims expressed in this article are solely those of the authors and do not necessarily represent those of their affiliated organizations, or those of the publisher, the editors and the reviewers. Any product that may be evaluated in this article, or claim that may be made by its manufacturer, is not guaranteed or endorsed by the publisher.
